# Aspects of Electrochemical Biosensors Using Affinity Assays

**DOI:** 10.3390/bios15030166

**Published:** 2025-03-04

**Authors:** Thor Pedersen, Leonid Gurevich, Nils E. Magnusson

**Affiliations:** 1 Department of Materials and Production, Aalborg University, Fibigerstræde 16, 9220 Aalborg, Denmark; tlvp@mp.aau.dk; 2 Biostrip APS, Lindevangsvej 10, 8240 Risskov, Denmark; 3 Medical Research Laboratory, Department of Endocrinology and Internal Medicine, Aarhus University Hospital, Palle Juul-Jensens Boulevard 165, 8200 Aarhus, Denmark

**Keywords:** biosensor, nanomaterials, electrochemistry, screen-printed electrodes, electrochemical labels, electrochemical biosensor, surface modification, immobilization, immunoassay

## Abstract

In recent decades, the utilization of biomarkers has gained increasing attention. The timely identification and quantification of proteins, nucleic acids, and small molecules associated with a medical condition, infection, or contaminant have become increasingly crucial across a variety of fields, including medicine, food safety, and quality/environmental control. State-of-the-art biomarker detection methods predominantly rely on standard immunoassay techniques, requiring specialized laboratory equipment and trained personnel. This impedes the broad commercial implementation of biosensors in, e.g., Point-of-Care (PoC) settings where ease of operation, portability, and cost-efficiency are prioritized. Small, robust electrochemical biosensors are a promising alternative for analyzing biomarkers in complex samples within PoC environments. Therefore, creating and designing optimized sensing surfaces, immobilization strategies, and efficient signal generation are crucial for improving biosensor systems, which in turn can have real-world impact. In the present paper, we reviewed common electrode types and geometries used in electrochemical biosensors and the immobilization approaches, discussed the advantages and drawbacks of different electrochemical detection methods, and presented different labeling strategies for signal generation and enhancement.

## 1. Introduction

The application of biomarkers has become a focal point of research and innovation, playing an increasing role in fields such as medicine, environmental monitoring, and food safety. Soluble biomarkers, which include proteins, nucleic acids, and small molecules, offer valuable insights into the diagnosis, progression, and treatment of diseases, as well as the detection of contaminants. The majority of current detection methods rely on traditional immunoassay techniques, which require sophisticated laboratory infrastructure, specialized equipment, and trained personnel. These requirements significantly limit the accessibility and scalability of such methods, particularly in PoC settings, where ease of operation, portability, and cost-effectiveness are essential. Small electrochemical biosensors are analytical devices involving recognition molecules such as antibodies, aptamers, antigens, DNA receptors, or enzymes in contact with a transducer [1]. An important benefit of biosensors is their significantly lower cost compared to standard laboratory methods. In addition, biosensors can be integrated into portable and easy-to-use devices. By integrating biological recognition molecules with electrochemical detection systems, these biosensors can achieve high sensitivity and specificity. Their compact size, rapid response time, and potential for low-cost manufacturing make them particularly well-suited for decentralized healthcare settings. As such, electrochemical biosensors offer a promising alternative for biomarker analysis in PoC environments.

Since the introduction of the amperometric glucose enzyme electrode [2], the most successful PoC devise for glucose monitoring, various biosensors have been marketed, mainly directed towards small molecules and ions, such as cholesterol, uric acid, glucose, and lactate. For a comprehensive list, see the reviews by Wu et al. and Labib et al. [3,4]. By contrast, the clinical utility of biosensors remains limited because of difficulties in integrating and miniaturizing biosensors into portable devices. In addition, the detection of large molecules, such as soluble proteins, requires several steps during measuring and signal amplification to achieve a detectable signal. This makes it more difficult to integrate them into PoC devices, highlighting the need to develop technical robust and scalable solutions for biosensor systems, especially for large-molecule applications. The development of optimized sensing surfaces, immobilization techniques, and signal amplification strategies is critical to enhancing the performance and real-world applicability of these systems.

This review explores the core components and methodologies of electrochemical biosensors, focusing on electrode types and geometry, immobilization strategies, and the use of labels for signal generation and enhancement. It explores the advantages and limitations of different electrochemical detection methods, while also considering their potential for practical applications in real-world settings. Particular emphasis is placed on screen-printed electrodes (SPEs) and thin-film electrodes, which offer cost-effective and scalable solutions for biosensor fabrication. The manuscript also examines emerging approaches, such as molecular imprinting and click chemistry, which have shown promise in improving receptor immobilization and biosensor stability.

By addressing these topics, this review aims to provide an overview of the state-of-the-art in electrochemical biosensors, shedding light on their transformative potential for PoC devices and beyond.

## 2. Electrodes

Electrodes are a fundamental part of any electrochemical biosensor. They typically take the form of macroscopic solid rod or disk electrodes, SPEs (also known as thick-film) or thin-film electrodes. Macroscopic solid electrodes are extensively used within electrochemistry; however, they are unsuitable for PoC biosensor applications as they are costly and bulky, incompatible with high-throughput modification, require large solution volumes, and are not intended for single use and require washing and cleaning between measurements. These limitations mean they are generally used for proof-of-concept and not practical biosensor applications [5,6].

### 2.1. Electrode Types

In comparison, SPEs and thin-film electrodes are a more attractive option for PoC biosensors. With these technologies, the reference or pseudo-reference electrode (RE), the counter-electrode (CE), and the working electrode (WE) can all be placed together on a single piece of substrate. Since their appearance in the 1990s, the application range of SPEs has continuously grown, owing to their cost-effectiveness, reproducibility, reliability, and scalability for mass production. The SPEs have shown to be incredibly versatile and can be adapted to various configurations and fabricated on several materials, as well as being amenable to modification with a wide variety of biological elements, such as DNA, enzymes, cells, and antibodies [7,8,9]. Table 1 contains a selection of SPEs and thin-film electrodes that are commercially available and examples of custom-made ones. We also refer the reader to [10,11,12,13] for additional publications from different suppliers/producers.

**Figure 1:**
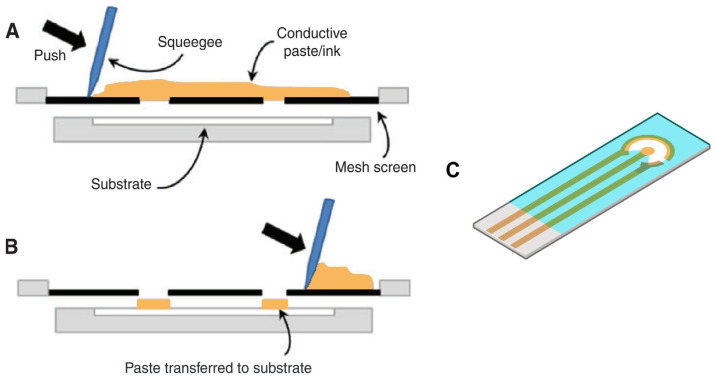
Illustration of screen-printed electrode fabrication process. (**A**) First, a conductive paste is placed onto the mesh screen, containing open and closed regions; (**B**) With a blade (squeegee), the paste is pressed through the openings in the mesh onto the substrate. This step is followed by screen-substrate separation and drying. (**C**) An example of a ready-to-use device (modified from [23]; used under CC BY 3.0).

SPE fabrication consist of a few steps, which can be summarized as (1) the design and selection of mesh/screen/mask, (2) the selection and preparation of conductive ink/paste, (3) the selection of appropriate substrate material, (4) layer-by-layer deposition of the ink(s), (5) the drying and curing of the electrodes, and (6) the deposition of an insulating layer. The mesh is what defines the electrode geometry and size, with a minimum feature size limited to a couple of hundreds of microns. For ink selection, there is a range of aspects to consider. Since the ink is a composite consisting of micro/nano-dispersed conductive material mixed with binders and organic solvents, its properties, such as its adhesiveness, conductivity, viscosity, as well as the type of binders and the solvents used, are important for both the fabrication process and functionality [6,7,8,9,14]. Many different types of substrates have been used for screen-printing, such as ceramic, alumina, glass, paper, plastic, and fabric [7,8,15,16,17]. The substrate must be compatible not only with the used inks but also with the drying and curing steps during fabrication. The layer-by-layer deposition is carried out by adding the ink to the mesh and having a blade (squeegee) move across the mesh to push the ink through the gaps and onto the substrate below, as illustrated in Figure 1A,B. After deposition, the substrate is dried and cured before the next deposition is performed. Several layers of different inks can be deposited and overlayed with each other, forming a complete circuit with the WE, CE, and RE. Finally, the electrode is coated with an insulating layer to limit the solution-accessible area [7,18,19,20]. For a more detailed review of SPEs, as well as their fabrication, ink composition, etc., see [21,22].

This fabrication process makes SPEs quite practical for PoC biosensors as their size and shape are easily customized and they have lower required sample volumes and a large degree of freedom for substrate materials and inks while still being economical. Nevertheless, the process does have some inherent variability, estimated to be close to or greater than 5% [24,25,26,27,28,29].

**Table 1:** Selection of screen-printed and thin-film electrodes.

Screen-Printed Electrodes			
Material	Produce/Supplier	Notes	Reference
Gold	DropSens	BT220, AT220, AT250	[30,31,32,33,34,35,36,37,38,39,40]
	BVT Technologies	BVT-AC1.W1.RS.Dw2	[41]
	PalmSens	Italsens	[42,43]
	Zimmer and Peacock	A-AD-GG-101-N	[44]
	Zensor R&D	X	[45]
	Micrux	X	X
	Gamry	X	X
	Pine Research	X	X
Carbon/graphite/graphene	DropSens	110, C110, 110CNT	[34,36,46,47,48,49]
	BVT Technologies	AC3.W4	[50]
	Micrux	S1PE	[51,52,53]
	PalmSens	Italsens	[54]
	Zimmer and Peacock	X	[55,56]
	Zensor R&D	TE100	[57,58,59,60]
	iGii	Gii-Sens (3D foam)	[61,62]
	Custom	X	[16,17,63,64,65]
	Conductive Technologies	X	X
	Gamry	X	X
	Pine Research	X	X
Thin-Film Electrodes			
Material	Producer/Supplier	Notes	Reference
Gold	DropSens	Interdigitated electrode	[66]
	Micrux	Interdigitated electrode and SE1-AuPT	[67,68,69,70]
	Custom-made	X	[71,72,73,74,75]
	Macias Sensors	PCB electrodes	X
	Conductive Technologies	X	X
	Zimmer and Peacock	X	X
Carbon/graphite/graphene	Custom	X	[76,77]

Depending on the desired properties, each individual electrode (WE, CE, RE) could be made using different inks. The WE and CE are typically made with the same conductive material in the ink, which is most often gold or carbon. Both materials offer good mechanical and chemical resistance, high conductivity, low electrochemical electron-transfer resistance, and easy surface modifications. Carbon inks are typically made up of graphite particles, but they can also contain graphene, carbon nanotubes (CNTs), and fullerene, which offer favorable properties such as increased conductivity and broad potential range. There are other useable materials, such as copper, platinum, palladium, nickel, and indium tin oxide, although their use is much more limited [7,18,19,78]. The RE is usually made with silver/silver–chloride inks. As a matter of fact, this type of RE is a pseudo-reference due to the ill-defined concentration of chloride ions at the surface, which leads to drifting potential [79].

A large variety of inks are commercially available; however, other than the conductive material, their formulation (binders, solvents, etc.) is not disclosed by the supplier. Furthermore, the inks can be altered by adding modified materials to improve their electrochemical performance. This includes materials such as inorganic, polymeric, carbon-based, and composite nanomaterials [6,7,18,19]. This poses a challenge as inks may not only differ in composition, but also in their drying and curing processes, which directly tie to the electrochemical performance of the SPE, including electron-transfer resistance and electrochemical active surface area, as well as the feasibility of subsequent modifications [9,18]. This was very well demonstrated by Kadara et al. [58], who electrochemically probed multiple commercial SPEs, which were shown to display different electrochemical reactivities despite nominally having the same conductive material (carbon). This difference in SPEs played a direct role in biosensor performance, as demonstrated in [40,60,74]. Kerr et al. [60] showed how the signal output changed depending on the commercial SPE used as a platform for the biosensor. In [40,74], it was found that one of the gold SPEs tested did not generate an electrochemical response, while other gold SPEs produced an electrochemical signal when subjected to the same protocols. This highlights the carefulness one must exercise when working with SPEs, as a developed protocol that works perfectly for one SPE may not work at all with another, even when both, e.g., are made of gold or even come from the same supplier.

An alternative to SPEs is thin-film electrodes. These are often fabricated using a combination of optical lithography and physical vapor deposition techniques (e.g., evaporation or sputtering), which yields highly uniform, durable, chemical and mechanical resistant electrodes [80,81]. Just like SPEs, these techniques can used on a variety of substrates like polydimethylsiloxane, glass, silicone, poly(methyl methacrylate), and polyethylene terephthalate that has been glycol-modified [71,82,83,84]. Due to the high resolution of thin-film technology, limited by the lithography technique, it is possible to create fully customizable, complex electrode geometries, including micro-electrodes, interdigitated electrodes, and multiplex arrays. Commercially available thin-film electrodes typically have a minimum feature size of a few microns, making it possible to miniaturize the electrodes and the overall sensor size. Due to the nature of the fabrication, pure conducting materials can be deposited without the need for any binders or solvents, which makes the electrodes well-suited to subsequent modifications. An added benefit of this is that the electrodes are completely interchangeable with a well-defined surface chemistry. However, the fabrication of thin-film electrodes involve multiple laborious and time-consuming steps, which often makes these electrodes more costly than SPEs.

### 2.2. Electrode Geometries

**Figure 2:**
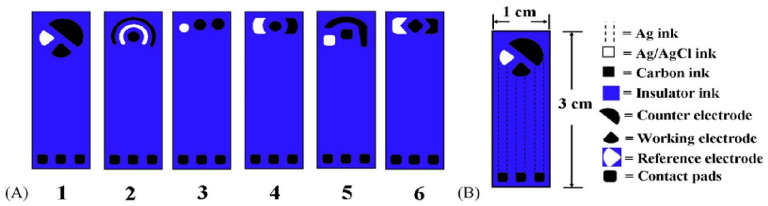
(**A**) Tested electrode configurations for SPEs made of carbon and (**B**) first configuration showing the sliver tracks and physical dimensions [87] (reprinted with permission from Elsevier).

As previously described, both SPEs and thin-film electrodes can have customized geometries, and this aspect can be exploited to some extent to improve the electrode’s electrochemical performance. For SPEs there are three geometric parameters to mainly consider: the WE area, the gap between the WE and CE, and the configuration of WE, CE, and RE. The WE area directly affects the current as all electrochemical phenomena are linked via the current density [85]. The gap between the WE and CE also plays a role, as was simulated and demonstrated by Roslan et al. [86]. They found that decreasing the gap led to an increase in the current density, which yielded better SPE performance. Lastly the geometry and placement of electrodes also plays an important role, as was demonstrated in [87]. They tested six different configurations of carbon SPEs using cyclic voltammetry (CV) with K3Fe(CN)6, see Figure 2, where the “classical setup” with the RE and CE on opposite sides of the WE (see Figure 2A and configuration 5), yielded the highest sensitivity and reproducibility. Another approach to the electrode configuration is the design from Zimmer and Peacock, where the electrodes are smaller and have a rectangular shape [88]. Adopting this configuration and placing the electrodes parallel to each other, makes a sensor better compatible with fluid transport as the electrodes will function as a fill sensor and have improved interaction with the fluid profile [89].

The fabrication of thin-film electrodes allows for smaller feature sizes and complex geometries, which can be taken advantage of. Designing WEs on the micron or submicron scale offers several advantages such as enhanced signal-to-noise ratio, lower ohmic losses (resistance), reduced capacitive effect due to the smaller surface area, surface diffusion changing from planar to radial increasing mass transportation to and from the electrode, and reduction in polarization time, which in turn lowers setup time and assay time [56,68]. Some of these advantages can even be utilized for SPEs if the electrodes can be produced with a small enough size, as was demonstrated in [56]. An additional benefit of the smaller electrodes is that overall size also decreases, which makes it easier to create electrode arrays that can be used for PoC biosensors capable of measuring multianalyte samples. A typical geometry design is the interdigitated electrodes. This design offers the possibility of measuring the capacitive changes in the space between the WE and CE and/or when molecular binding events occur on the electrode surface [66,90]. In addition, the design can be used for redox cycling, which was shown to improve biosensor performance [91,92]. By finetuning the geometric parameter such as electrode width, gap size, number of fingers, and height it is possible to improve upon the electrochemical performance [67,91,93,94].

## 3. Immobilization

Immobilization of a receptor (DNA, enzymes, antibodies, and cells) onto the electrode surface is a critical step for electrochemical biosensors. It directly affects the obtainable signal from specific binding events while influencing stability and applicability of the biosensor. It is important to select an appropriate immobilization method that retains activity of the receptor, ensures suitable receptor orientation to facilitate binding, and provides a good density of receptors on the electrode surface [95,96]. Before performing any immobilization or surface modification, it is essential to clean the electrode surface either mechanically and/or electrochemically for achieving good electrochemical performance and high immobilization density [97,98,99].

**Figure 3:**
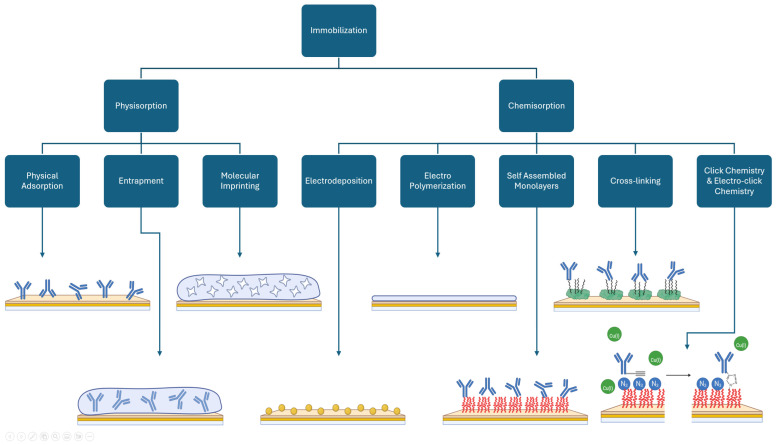
Overview of various immobilization/modification methods including graphical representations. From left to right; physical adsorption, entrapment, molecular imprinting, electrodeposition, electropolymerization, Self assembled monolayers, cross-linking, and click chemistry and electro-click chemistry.

Multiple techniques can be used to accomplish the immobilization, as presented in Figure 3, which can be split into two categories, based on the interactions involved: physisorption and chemisorption. Physisorption is based on weak interactions such as van der Waals forces while chemisorption is based on strong interactions like covalent bonds [96,100]. It is common to use or combine multiple techniques for immobilization to modify the surface, create functional groups, and achieve better reproducibility, stability, and performance [41,46,47,54,61,101].

### 3.1. Physisorption

#### 3.1.1. Physical/Passive Adsorption

This type of immobilization is the simplest way of attaching a receptor onto the electrode surface. The attachment is due to weak interactions such as electrostatic and hydrophobic interactions, hydrogen bonding, and van der Waals forces. With a sufficiently high concentration it is possible to form a layer on the surface with this method. Many factors influence the quality of the adsorbed layer such as electrode surface contaminations, blocking of binding sites due to sub-optimal orientation of the receptor, and partial denaturation of the receptor. The adsorption process yields random orientation of receptors and has a possibility of spontaneous desorbing upon a change in temperature, ionic strength or just with time. This leads to lower sensitivity, reproducibility, and stability making it a less efficient biosensor. However, this method is still commonly used for due to its non-destructive process, low cost, and ease of implementation [14,96,100].

In many cases, adsorption serves as an intermediate step in the immobilization process. Instead of adsorbing the receptors directly on the surface, materials with a strong affinity to the surface, such as pyrene compounds, carboxymethyl dextran, gold nanoparticles (AuNPs) etc., are adsorbed first. Then, depending on the functional group available, the receptor can be adsorbed on top or covalently bound by, e.g., cross-linking or carbodiimide chemistry [54,56,102,103]. Another option is to coat the surface with proteins possessing a specific affinity (lock-key interaction) and then use it to bind and orient a receptor, e.g., Protein A or G and (Strept)avidin-biotin [34,44,103]. Elshafey et al. [104] and Kausaite-Minkstimiene et al. [105] used Protein G to enhance oriented binding of antibodies resulting in an increase in the binding capacity of the analyte. In [105] the binding capacity of oriented and randomly bound receptors were compared using surface plasmon resonance. It was observed that when the receptor was oriented the signal was 11 times higher than for randomly bound.

#### 3.1.2. Entrapment

An alternative approach is entrapment of a receptor in a polymer matrix, e.g., hydrogel, a sol–gel, or chitosan. After mixing the receptor with the building blocks of the polymer, the polymerization/gelation can be initiated by either (1) a chemical reaction or (2) a change in environmental conditions such as temperature or pH, facilitating the entrapment of the receptor. The entrapment method offers several benefits in comparison to passive adsorption such as chemical inertness, mechanical rigidity, higher thermal stability, and lowering of denaturation while still being simple to prepare. An added benefit is that the entrapment can be done at low temperatures, and it yields transparent gels that can have their porosity finetuned [96,106,107]. However, if the porosity of the matrix is not sufficient it will cause diffusion limitations, which in turn will lower the sensitivity and thereby the biosensor performance. On the other hand, if the pore size is too large it will cause leaching of the receptor [106]. Entrapment, for instance, was utilized by Lipińska et al. [108] to immobilize glucose oxidase in a chitosan matrix onto a nanostructured titanium foil with AuNPs creating a glucose biosensor with good sensitivity that was resistant to a range of interferents. For more information on this method, we refer readers to [106] for an in-depth review.

#### 3.1.3. Molecular Imprinting

Although not technically classified as an immobilization technique, molecular imprinting yields a layer with the same recognition and binding functions as an immobilized receptor.

This method is similar to entrapment, where one of the main differences is that the biological element is trapped in the former while only partially bound in the latter. The idea of imprinting is to create a cavity with a size and chemical interaction that are complementary to the analyte, meaning that if a sample contains the analyte, it will bind selectively and specifically to these cavities. To accomplish this the analyte is initially introduced into the polymer matrix followed by cross-linking and analyte removal. The process of forming cavities can be a significant challenge as multiple factors need to be controlled so that the matrix cross-links enough to form the correct shape but without locking the analyte in the cavity, which would render it unusable.

The method has many of the same advantages as entrapment such as robustness and chemical inertness. In addition, as the method does not require immobilizing receptors or analytes, it circumvents the problem of receptor/analyte denaturation while providing equivalent or higher sensitivity and affinity to the target. Addition of nanomaterials like graphene oxide (GO), AuNPs, etc. to the polymer matrix have shown a positive effect on catalytic activity, biocompatibility, and conductivity improving the electrochemical biosensor performance [109,110,111,112]. As an example, Özcan et al. [113] made use of imprinting to create selective cavities on a glassy carbon electrode that was modified with a nanocomposite material consisting of multi-walled CNTs functionalized with graphene quantum dots. Using the nanocomposite material the biosensor showed improved electrochemical characteristics (charge transfer resistance and peak separation voltage) and sensitivity. Together with the imprinting method the biosensor showed good selectivity with minimal signal from interferents, repeatability, and stability. For more information on this method we refer the reader to [111,112,114].

### 3.2. Chemisorption

As previously mentioned, chemisorption typically equates to covalent binding which is one of the most widely used strategies for binding receptors to the electrode surface. However, unless the correct functional groups are initially present on the receptor or on the electrode, it will be necessary to do some modification. The functional groups typically used for covalent binding are carboxyl, amine, and thiol groups where the binding will depend on various factors like pH, temperature, time, and the functional group on the substrate and receptor. However, for commonly used metal electrodes the binding chemistry is rather limited often only allowing for thiol groups to bind directly to onto the unmodified gold or other coinage metal surfaces [115]. To broaden the available chemistries for covalent immobilization of a receptor and reduce nonspecific binding, surface modifications via adsorption, electrodeposition, self-assembled monolayers (SAMs), cross-linking, and electropolymerization are needed.

#### 3.2.1. Electrodeposition

Modifying the surface from solution via adsorption is the simplest and can be used with a variety of compounds, proteins, and nanomaterials as previously mentioned. Here nanomaterials are of especial interest as these can be customized beforehand and offer specific functional groups for immobilization while lowering electron-transfer resistance, increasing electrode surface area, enhancing sensitivity, and improving electrochemical biosensor performance [115,116,117]. Instead of adsorbing the nanomaterial to the electrode surface one can actively utilize the electrode itself to incorporate or deposit the material. This will help introduce the functional groups needed for the immobilization of the receptor. The technique is called electrodeposition, and using it to deposit nanomaterials is not only faster but also offers greater control over the surface modification [115,116,117,118,119,120]. A different modification method, which is similar to electrodeposition, is electropolymerization.

#### 3.2.2. Electropolymerization

Electropolymerization typically uses conductive polymers to form a film on the electrode surface. This method offers simple control over film thickness, morphology and can have their chemical-, electrical-, and structural properties finetuned making them well suited for electrochemical biosensor applications [109,121,122]. Depending on the functional groups available a variety of covalent bonds can be made, in addition, the method is often used to entrap or imprint the analyte. Wang et al. [123] demonstrated how both electrodeposition and electropolymerization could be used together to create a biosensor. First a glassy carbon electrode was cleaned and then submerged into HAuCl_4_ solution where CV was performed to form and deposit the AuNPs onto the electrode surface. After the modification, the electrode was exposed to a multifunctional peptide that binds to the AuNPs via a thiol bond, which was followed by electropolymerization of 3,4-ethylenedioxythiophene monomer to form a conducting thin film. By using electropolymerization to form and control the thickness of the film, improvements to both stability and conductivity were observed. Combing this with the multifunctional peptide they observed better performance compared to 6-mercaptohexanol SAM with a current drop of 3% and 75% respectively, due to nonspecific binding from exposure to undiluted human serum. For a more in depth review on these two methods we refer to [109,110,121,122,124].

#### 3.2.3. Self Assembled Monolayers

The thiol-gold interaction has not only been exploited to bind molecules directly to the surface but to also form SAMs. Typically, alkanethiols are used for SAMs, which consist of a thiol group, an alkyl chain, and an additional functional group. These molecules exist with a plethora of functional groups available offering excellent customizability and versatility, which together with variable alkyl chain length gives the user even more options for immobilization and finetuning of the surface properties. A common method to facilitate the thiol binding and form the SAM on a gold surface is by exposing it to a solution of alkanethiol for an extended period of time. Depending on the time the surface is exposed, the layer has different characteristics in terms of density and, if the surface allows for it, order. One of the driving forces behind the process is the van der Waals interaction between the alkyl chains. This force plays an important role when forming the layer slowly as it helps in forming higher packed monolayers. The functional group also plays a role in the SAMs formation: if the group is charged or partially charged it will lead to a repulsion between these groups, which may hinder the formation of a densely packed layer [125,126]. One of the most common functional groups for SAMs is carboxylic acid. Carboxylic acid groups can be easily activated by using N-hydroxysuccinimidyl (NHS) esters and/or carbodiimides like 1-ethyl-3-(-3-dimethylaminopropyl) carbodiimide (EDC), which can subsequently be reacted with primary amines, forming a covalent bond. With this approach, any receptor containing a primary amine group can be attached to the SAM. It is even possible to invert the order and have the SAM with a primary amine as the functional group and then activate the carboxylic acid on the receptor [127].

An important benefit of SAMs is its anti-fouling properties. This aspect is crucial as reducing the non-specific binding in biosensors directly ties together with its performance. Therefore, having a method that helps alleviate this problem from the beginning makes it highly desirable. To obtain this type of effect it is important to finely control the layer and its properties, which as previously mentioned can be done by choosing certain functional groups and chain lengths. Although it can also be achieved by making mixed SAMs, containing two or more different thiol compounds to form the SAM. By using multiple thiols one can introduce multiple functionalities, increase layer density, promote specific binding and reduce non-specific binding, and tune various surface properties such as wettability. One example of this was performed by Ferreira et al. [31] where they compared two different strategies for immobilization: one using pure 6-mercapto-1-hexanethiol SAM and the other using a mixed SAM of 6-mercapto-1-hexanethiol and hexanedithiol. They found that the mixed SAM layer yielded a lower limit of detection (LOD) and reduced nonspecific binding. In addition to alkanethiols other options exist that can be used for SAMs or mixed SAMs like poly(ethylene glycol) and oligo(ethylene glycol), which have a positive effect on anti-fouling, aromatic compounds, e.g., thiophenol, p-biphenylthiol, and p-terphenylthiol or N-heterocyclic carbenes [127,128]. A possible shortcoming of this method is long term stability as prolonged exposure of the SAM layer to ambient environmental conditions, more specifically UV irradiation, causes the sulfur head groups to undergo oxidation, which hampers the functionality of the surface. Another issue with SAM formation is reproducibility. As the process is a spontaneous self-organization of molecules it can be affected by multiple factors like temperature, solvent, water content, exposure time, and SAM solution concentration, which makes it almost impossible to fully control [126]. For a more comprehensive in depth explanation on this topic see reviews [126,128].

#### 3.2.4. Cross-Linking

Another method of covalently binding the receptor is via cross-linking. It makes use of multi-functional reagents that acts as a linker between the receptor, e.g., a non-functional protein like bovine serum albumin, which can then be physiosorbed to the surface as demonstrated by Rafat et al. [34]. There exist a variety of reagents like glutaraldehyde, bis(sulfosuccinimidyl)suberate, and imidoesters, which can be classified into either homobifunctional or heterobifunctional cross-linkers depending on if the reactive group is identical or different from each other. To achieve optimal cross-linking it is important to consider factors like pH, temperature, time, linker concentration and ionic strength. Although the method offers advantages such as strong binding of the receptor and increased stability towards heat and organic solvents it also has some disadvantages. As the cross-linking reaction typically is not site specific and will react with any appropriate groups it leads to the formation of a variety of cross-linked complexes some being the desired receptor-to-x and others being, e.g., receptor-to-receptor complexes. Depending on the degree and location of cross-linking this can cause denaturation, aggregation, and/or activity loss of the receptor. However, the extent of this problem can be somewhat mitigated using optimal cross-linking conditions albeit finding these conditions can be troublesome [107,129]. An example that actively utilizes the aggregates formed via cross-linking is cross-linked enzyme aggregates (CLEAs). Šulek et al. successfully created CLEAs by cross-linking horse radish peroxidase (HRP) while retaining 83% activity compared to free enzyme, which could potentially be used for biosensor applications as, e.g., an enhancement tool [130].

#### 3.2.5. Click and Electro-Click Chemistry

A powerful yet simple approach that can be implemented to immobilize the receptor is click chemistry. Probably the most well-known type of click chemistry is the cycloaddition catalyzed by Cu(I) between an alkyne and an azide (CuAAC), which was first introduced in 2002 by Sharpless et al. and Meldal et al. [131,132]. Other types also exist such as strain-promoted alkyne-nitrone cycloaddition, nucleophilic substitutions in particular ring-opening of strained heterocyclic electrophiles (e.g., epoxides, aziridines, aziridinium ions, and episulfonium ions), carbonyl reactions of the non-aldol type, and addition reactions to carbon-carbon multiple bonds [133,134,135,136]. The attractiveness of this methods stems from its high yields, minimal side reactions and by-products, retention of receptor activity, bioorthogonality, and high selectivity while being applicable in a range of different solvents, pHs, and temperatures [133].

An interesting aspect of the CuAAC and electrochemical biosensors is the use of copper(I) as a catalyst. It can be introduced by adding Cu(I) salt or by reducing Cu(II) ions. In particular, it is possible to generate the Cu(I) ions electrochemically at the electrode surface from Cu(II), which is also known as electro-click chemistry. This allows for controlled local generation of catalyst, meaning that a receptor can be selectively immobilized onto the desired surface. This allows for unique customization as the immobilization is localized to each electrode surface, which can be turned on or off making it an ideal method for modifying electrode arrays [48,84,137,138]. Svalova et al. [139] made use of this technique to attach an NHS functional group that was later used to covalently immobilize antibodies. They started by forming a film of polyvinylbenzylazide on the electrode surface via electropolymerization that simultaneously also formed and incorporated copper NPs. Adding propargyl-NHS onto the electrode and applying a voltage, which oxidizes the copper NPs to the Cu(I) catalyst initiating the click chemistry and forming a covalent bond, thereby attaching an NHS functional group. NHS was then used for immobilization of antibodies. Using this approach the group noted that they could make a near electrode reaction that enabled them to reduce immobilization time of the antibody. For more information on both click chemistry and electro-click chemistry see reviews [101,135,140].

## 4. Electrochemical Techniques

**Figure 4:**
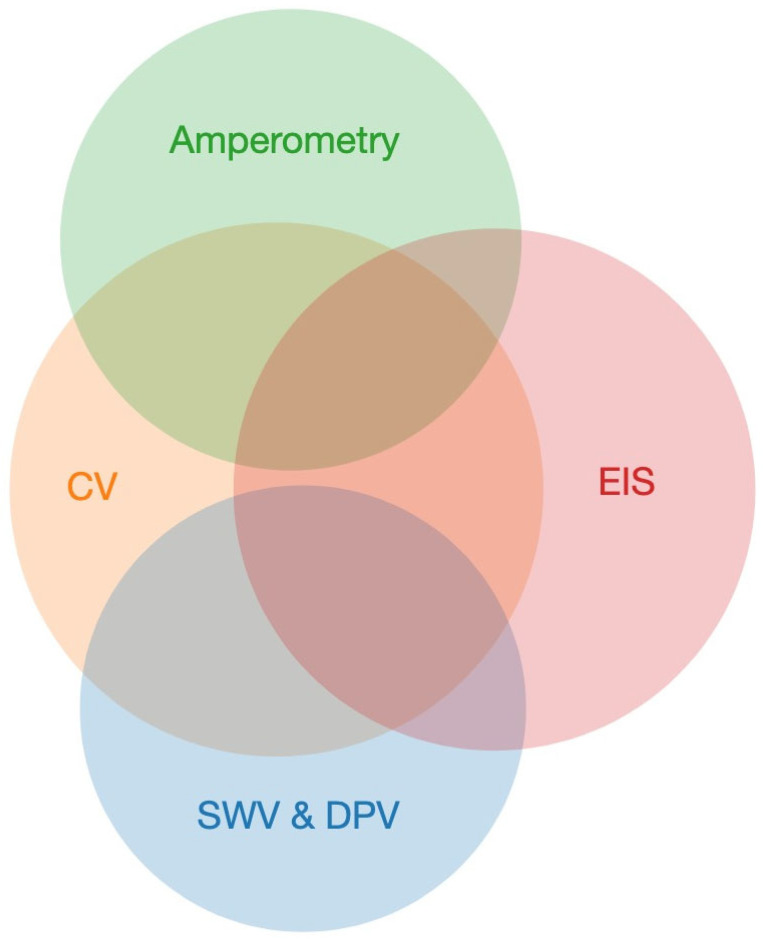
Proportional Venn diagram showing relative frequency of common electrochemical techniques appearing in biosensor publications during the last 10 years (1 January 2015–1 January 2025), excluding reviews. Overlaps represents papers where two or more techniques were used; overlap between SWV&DPV and Amperometry is ca. 20% but not shown due to geometrical reasons.

CV and electrochemical impedance spectroscopy (EIS) have emerged as the most commonly utilized techniques in electrochemical biosensors over the last ten years, as illustrated in Figure 4. This can be related to simplicity of CV and the wealth of information it can provide on the electrochemical reactions and utility of EIS in quantitative characterization of the electrode-solution interface. Another factor is wide-spread availability of inexpensive computer-controlled potentiostats, which offer both EIS hardware capabilities and software tools for efficient fitting of the obtained spectra. They are closely followed by amperometry and pulse voltammetry techniques—differential pulse voltammetry (DPV) and square wave voltammetry (SWV). Another interesting insight is that multiple techniques are commonly used in combination as illustrated by the overlapping of circles in Figure 4.

As the most common techniques mentioned above involve measuring of current under varied voltage applied, distinction of Faradaic and non-Faradaic responses is essential for proper interpretation of the experimental data (see, e.g., a recent discussion in [141]). A Faradaic process is generally related to charge transfer across the electrode/electrolyte interface. In this respect, the Faradaic current represents the actual electrochemical reactions, where the obtained *i*-*V* curve (polarization curve) can be related to a certain interplay of reaction and mass transfer kinetics. On the other hand, a non-Faradaic process represents charging of a capacitor formed by the electrode and solution and includes contributions from all the processes where charged species are accumulated at the interface without changing their redox state, such as double-layer capacitive response and adsorption. Due to this, non-Faradaic processes cannot maintain continuous direct current across the device but instead lead to a transient current appearing every time the electrode potential is changed. The non-Faradaic processes are better described by σ-V curve (surface charge vs. voltage applied). Below we will describe the interplay of Faradaic and non-Faradaic currents in the electrochemical techniques commonly used in biosensors.

**Figure 5:**
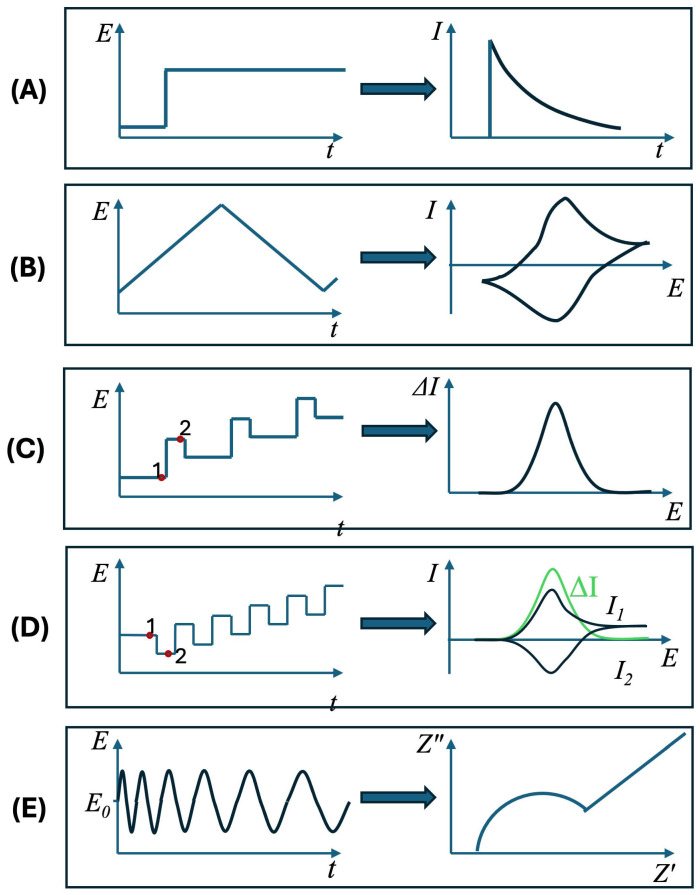
Common measurement techniques in electrochemical biosensors. Left panels show the input modulation; right panels are a schematic representation of the obtained signals. (**A**) Chronoamperometry; (**B**) cyclic voltammetry; (**C**) differential pulse voltammetry (DPV); (**D**) square-wave voltammetry (SWV); (**E**) electrochemical impedance spectroscopy. Red dots show the points where currents *I_1_* and *I_2_* are measured in DPV and SWV methods; *ΔI* denotes the difference between the currents.

Amperometric sensors typically employ chronoamperometry (Figure 5A), which is based on measuring the current that arises upon increasing the WE potential from a value where no electrochemical reaction occurs to a value where the concentration of electroactive drops to zero on the electrode surface [142]. While the potential step unavoidably creates a non-Faradaic current response, it exponentially decays with the characteristic time ($\tau =R_{S}\cdot C_{dl}$) defined by the solution resistance and double-layer capacitance, which is typically below a millisecond. Therefore, skipping the first approximately 100 ms of the obtained *i-t* curve is usually sufficient to exclude non-Faradaic contribution and fit the remaining curve to the Cottrell equation. For most purposes, it is sufficient to use the fact that the amplitude of the current in the Cottrell equation is directly proportional to the bulk concentration of the involved electroactive species at any point in time and, for instance, average the measured current over a certain time range [74]. Amperometry is commonly used for signal readout in enzymatic and enzymatically labeled biosensors. In this case, typically, the same electroactive species are involved in both enzymatic redox process and electrochemical processes on the electrode surface. The measured electrochemical current can be used to either determine the concentration of the electroactive species, or if it is fixed, the amount of the immobilized enzyme. It should also be noted that to achieve high sensitivity in a sensor, it is essential to ensure the fast kinetics of the electrochemical reaction on the electrode surface. In many cases, this can be achieved by modifying the electrode with nanomaterials such as reduced GO, metal NPs, and CNTs. For instance, in [143], chronoamperometry was employed to measure catechol and other phenolic compounds using laccase or tyrosinase as enzymes, while in [144], cholesterol was measured using cholesterol oxidase and cholesterol esterase. In [74,145], HRP was used as a label in combination with respective antibodies to measure concentrations of *Brettanomyces bruxellensis* yeast cells in wine and various biomarkers, respectively. An important advantage of amperometric sensors is their simplicity in terms of sensor design, measurement protocol, and hardware implementation, combined with their excellent sensitivity. This makes them the method of choice for personal glucose sensors [146]. On the other hand, the method is prone to interference from compounds that can be oxidized or reduced within the same potential window and surface degradation, which can limit the measured current.

CV (Figure 5B) involves a linear scanning of the WE potential using a triangular waveform [142,147]. The technique provides wealth of information on the electrochemical reaction—its formal potential, the concentration of electroactive species, the reversibility and kinetic parameters of the reaction, the stability of electroactive species in reduced and oxidized forms, etc.—and is thds’us not only used for sensor operation but also for characterization and optimization purposes. Most of the studies of electrochemical biosensors, as a matter of fact, include CV measurements in combination with other methods, as can be seen from Figure 4. As compared to amperometric sensors, if multiple electroactive components are present, certain features at different potentials are observable with CV. This, however, relies on the detection of small peaks and correct determination of their peak current and peak position. For this, it is essential to separate Faradaic and non-Faradaic currents in the obtained CV curves, which is commonly achieved by subtracting the background slope. The procedure is, however, sensitive and might create substantial uncertainty in both peak current and position, particularly at high scan rates. Modern computational processing methods such as principle component analysis, partial least squares regression, and artificial neural networks therefore make CV a method of choice for the analysis of complex multicomponent systems, such as tea, amino acid mixtures, or beer [148,149,150].

DPV (Figure 5C) and SWV (Figure 5D) are the most commonly employed members of pulse voltammetric techniques. The overall idea of these techniques is that voltage is varied in small steps and current measurements are performed with a certain delay, typically about 50 ms, after the voltage step. In this way non-faradaic (charging) currents related to charging the double-layer capacitor are largely suppressed. Furthermore, in both DPV and SWV, the current is measured twice, after forward and reverse pulse fronts, leading to a differential measurement with even stronger suppression of charging currents. The method can be applied either directly to reduction/oxidation of the analyte in question or to a redox probe. In the latter case, changes in the surface properties upon binding the analyte are measured. Liu et al. [151] used this approach to design a sensor for cortisol in saliva, suitable for PoC environment. DPV with ferro-/ferricyanide redox probe was used to detect changes in carbon SPE modified with AuNP/MoS_2_/AuNP layer functionalized with anti-cortisol antibodies. This yielded a low detection limit for cortisol in sub-nM range, even when using a portable smartphone-based system with SPE electrodes. An example of direct electrooxidation of analyte species can be found in Sakthivel et al. [152]. The authors showed well-defined DPV peaks, corresponding to the individual component in a mixture of ascorbic acid, dopamine, and uric acid in a mixture with a LOD in hundreds nM range. As with other techniques relying on current measurement, it is essential to maximize both the rate of electrochemical reaction and the electrode surface area, to achieve better resolution and LOD. In this case it was achieved by depositing carbonized electrospun fibers decorated with AuNPs on a commercially available carbon SPE.

EIS (Figure 5D) is performed by applying a small-amplitude sinusoidal excitation and measuring the induced current, amplitude and phase [142,153]. Based on this, complex impedance is calculated, which is typically plotted as the so-called Nyquist plot with the imaginary (Z″) and real (Z′) parts of the impedance along the vertical and horizontal axes, respectively. From this graph an equivalent electric circuit can be constructed. In terms of separation of Faradaic and non-Faradaic currents, this represents an important advantage of the technique as various capacitive and resistive contributions, related to different physical phenomena: double-layer at electrode-solution interface, charge transfer, diffusion, adsorption and stray capacitances can be assigned to the circuitry components and separately determined. This is why EIS became a routine method, together with CV, to characterize the electrode surface and modification/immobilization procedures. Typically, EIS measurements are performed across the electrode-solution interface, a well characterized redox probe (ferro-/ferricyanide or ferrocene) is added to the solution and the experiment is carried out near the probe’s formal potential. This approach was implemented in aptamer-based impedance biosensor for detection of doxorubicin demonstrated by Bahner et al. [154]. The authors measured an increase in charge transfer resistance for ferro-/ferricyanide redox probe upon binding of doxorubicin to the aptamer layer. The sensor showed linear behavior in the doxorubicin concentrations range between 31 nM and 125 nM and LOD of 28 nM. On the other hand, EIS measurements can also be performed in a planar geometry between interdigitated electrode fingers. An recent example of this approach can be found in Panagopoulou et al. [155]. They demonstrated the detection of Pb^2+^ and Cr^3+^ ions using DNAzymes disassembling into smaller fragments in the presence of the ions. DNAzymes were attached to a PtNP film deposited on top of microfabricated gold interdigitated electrodes. Exposure to the analyte ions led to an increase in inter-electrode impedance with LOD of 0.8 nM and 10 nM for Pb^2+^ and Cr^3+^, respectively.

## 5. Labels and Surface Enhancements

An integral part of electrochemical biosensors is the usage of labels. The use of labels can be summarized into two main functions: (1) enhancement of electrochemical properties, e.g., surface area, electron transfer, conductivity, and/or (2) introduction/generation of electroactive species, e.g., redox probes, enzymes, catalytic particles. Depending on the label, it will cater towards specific electrochemical detection methods, meaning that not all labels are compatible with various detection methods. Therefore, selecting an appropriate label can have a profound impact on the biosensor and its performance. In addition, many of the labels can also be used to enhance the electrode surface to improve the electrochemical properties.

### 5.1. Metallic Nanoparticles

Metallic NPs have gained significant attention as labels in electrochemical biosensors due to favorable properties like electron transfer efficiency, catalytic activity, high surface-to-volume ratios, biocompatibility, and customizability. Although one of the drawbacks of metallic NPs is electrostatic stabilization, as they are susceptible to changes in ionic strength and pH, which leads to aggregation and precipitation. Therefore, their usage is limited when working with biological samples, unless appropriate modification of the NP surface is done to stabilize it, e.g., adsorbing receptors or/and coating with polymers [156].

One of the most extensively studied metallic NPs is AuNPs. They possess excellent biocompatibility, electron transferability, customizable surface modifications, and straightforward immobilization of receptors/proteins while being easy to synthesize [107,156]. These properties are leveraged in many electrochemical biosensors by modifying the electrode surface as previously mentioned in Section 3.2.1, or by combining it with other nanomaterials, e.g., graphene and CNTs [157,158]. AuNPs can be used as a label by modifying it with elements that are electroactive or can produce electroactive elements. In both cases, the AuNP functions as a carrier allowing for multiple elements to be attached per binding event on the sensor surface causing an increase in sensitivity.

**Figure 6:**
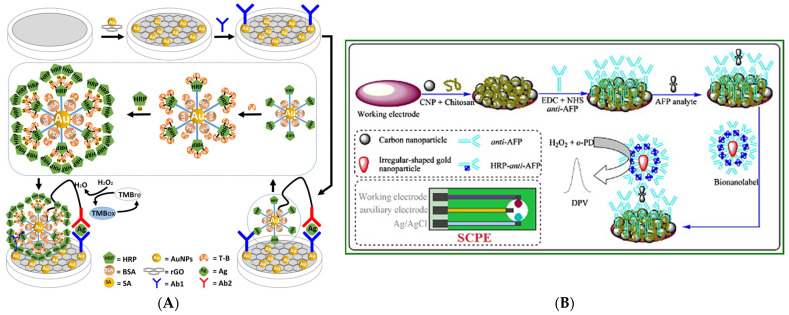
Schematic representation of the biosensor build-up allowing for the attachment of multiple HRPs per binding event in a sandwich assay: (**A**) A dendrimer-like structure with multiple HRPs on a single AuNP by Liu et al. [159] and (**B**) usage of irregularly shaped AuNP with coated with HRP-labelled antibodies by Tang et al. [160] (reprinted with permission from Elsevier).

This was demonstrated by Liu et al. [159] and Tang et al. [160] who immobilized an antibody and HRP to AuNPs. The complexes were then used to form a sandwich together with the antibody on the electrode and the analyte, as shown in Figure 6. In these examples, the purpose of HRP was to generate electroactive species from 3,3′,5,5′-tetramethylbenzidine (TMB) and o-phenylenediamine, which were detected via amperometry and DPV, respectively. Both observed that the use of AuNPs significantly improved the electrochemical response compared to when it was not used. Liu et al. also highlighted how it was possible to further improve the response by modifying the complexes with tyramine labeled biotin and streptavidin labeled HRP, which led to even more HRPs being bound per binding.

Other metallic NPs have been utilized in biosensors like silver NPs [161,162], platinum NPs [163], palladium NPs [164], copper NPs [165], by using them in a similar fashion as AuNPs or to achieve an enhancement. This is possible due to their characteristics such as customizability, catalytic activity, conductivity, and biocompatibility [107]. Some even use hybrid nanomaterials as labels for the enhancement, which can yield additional benefits depending on the combination of materials [166,167].

A subcategory of metallic NPs is metal oxide NPs. They can be made of a variety of metals where some of the more common metal oxides are zinc oxide (ZnO), titanium oxide (TiO_2_), iron oxide (Fe_2_O_3_), manganese oxide (MnO_2_), copper oxide (CuO), nickel oxide (NiO), and cobalt oxide (Co_3_O_4_). In general, these exhibit excellent electronic, catalytic, magnetic, chemical, mechanical, and optical properties [168,169]. Here the catalytic properties have garnered especial interest as NPs with this property could be used as a substitute for enzymes. These types of NPs are also known as nanozymes or artificial enzymes. Exchanging the enzymes with nanozymes can yield several advantages such as better stability, resistance toward external influences, and by controlling size/composition the activity can be adjusted to improve enzyme-substrate kinetics (K_m_ and V_max_) [170,171,172,173].

**Figure 7:**
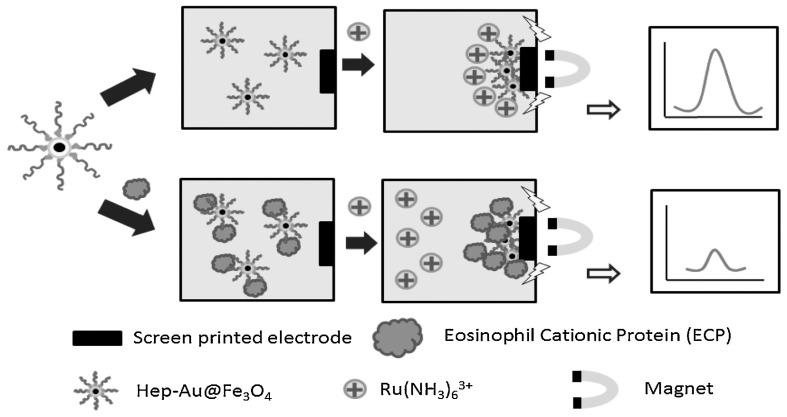
Representation of the electrochemical sensor principle designed by Lee et al. [175]. Magnetic NPs coated with heparin are used to capture the analyte (ECP) which are manipulated with a magnet onto the electrode surface. The SWV signal observed from the redox probe is inversely proportional to the analyte concentration (reprinted with permission from Elsevier).

Another attractive part of metal oxide NPs is the magnetic properties. The most common magnetic NP is Fe_2_O_3_ or Fe_3_O_4_ due to it simple preparation process, controllable size, and biocompatibility [174]. It is also possible to incapsulate the NPs in a polymer matrix to create larger magnetic beads, in either case they can be combined with electrochemical biosensors to create unique setups. Lee et al. [175] designed and tested such a system. They created a biosensor to detect eosinophil cationic protein using gold covered Fe_3_O_4_ NPs that were modified with heparin. When exposing the modified NPs to the protein it would adsorb to the heparin and when applying an external magnetic field, the NPs would be concentrated onto the electrode surface, as shown in Figure 7. Then depending on the amount of protein adsorbed onto the NPs the current would decrease when measuring with SWV. The utilization of magnetic NPs showcase how electrochemical biosensors could be created without requiring any modification or immobilization of receptors onto the electrode, thereby simplifying many of the fabrication steps. This approach could even be combined with other NPs creating a dual-particle configuration, that could be used for further enhancement.

We refer the reader to these reviews on NPs [176], metal oxide NPs [169], and magnetic NPs [177,178] for additional information.

### 5.2. Carbon Materials

CNTs are cylindrical nanostructures formed by rolling up one or several graphene sheets into single-walled or multi-walled tubes. These nanomaterials have several advantageous properties such as large surface area, flexibility, thermal and chemical stability, high conductivity, and high electron-transfer capability, which makes them well suited for electrochemical biosensors [107,156]. They possess a high surface-to-volume ratio due to the diameter being on a nanoscale while the length is on the microscale. In addition, the large surface area of CNTs can be utilized to increase the quantity of immobilized receptors and facilitate the electron transfer, which would improve the electrochemical biosensor’s performance by widening the measurable range and increase the signal response [156]. One of the main disadvantages of CNTs is that the synthesis process is complicated and requires skilled operators to ensure uniform performance and electrochemical characteristics. As there is little to no structural control at the atomic level it becomes a significant challenge to control the size of the CNTs as well [179]. Another disadvantage is the insolubility of CNTs, which significantly limits their use in biosensors. Although this can be partially mitigated by forming hybrid nanomaterials or modifying the CNTs with chemical groups or biological molecules [180]. CNTs or hybrid nanomaterials containing them are typically used to modify the electrode surface improving various properties, e.g., conductivity and electron transfer resistance, as previously mentioned in Section 3.2.1.

**Figure 8:**
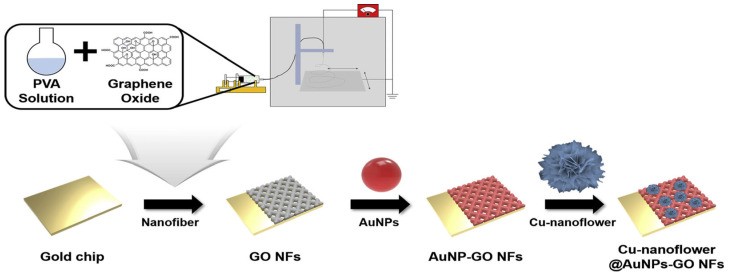
Schematic illustration for the fabrication of Cu-nanoflower and AuNPs-GO NFs-based electrochemical glucose nano-biosensor [182] (reprinted with permission from Elsevier).

Graphene is a two-dimensional nanomaterial with honeycomb lattice that has gained significant attention and is a promising material for electrochemical biosensors. This is largely due to their high specific surface area, high conductivity, flexibility, and possibility of customization. However, due to its hydrophobic nature it is problematic to dissolve in water [119,181]. Graphene also exists in oxygenated forms such as GO and reduced GO, which have become important materials for electrochemical biosensors. These oxidized derivatives contain various oxygen functional groups such as carboxyl, hydroxyl and epoxy, which gives them a certain advantage. Due to the presence of these functional groups, it makes the derivatives strongly hydrophilic allowing them to be more easily dissolved in aqueous solutions while also introducing chemical groups that can be used for modification and immobilization. These reactive oxygen groups are also used to make hybrid nanomaterials by integrating other materials like AuNPs, metal oxide NPs, and quantum dots, to further enhance the performance of the electrochemical biosensor [181]. Baek et al. [182] demonstrated how GO together with AuNPs and copper nanoflowers coated with HRP and glucose oxidase could be used as an electrochemical glucose sensor. They created nanofibers made of polyvinyl alcohol and GO via electrospinning, which were then decorated with AuNPs followed by the adsorption of copper nanoflowers. Figure 8 illustrates the biosensor fabrication process. They observed that the addition of GO nanofibers enhanced the electrochemical properties of the electrode surface, which could be further improved by the decoration of AuNPs. In addition, the AuNP-GO nanofibers had a synergistic effect with the copper nanoflowers for the detection of glucose. For additional information on CNTs, graphene and other carbon nanomaterials we refer the reader to [115,179,180,181,183].

### 5.3. Enzymes

One of the most common labels used in electrochemical biosensors is enzymes where some of the more widely used include glucose oxidase, HRP, alkaline phosphatase, and tyrosinase [184,185]. This is owed to the enzymes’ properties of high specificity/selectivity, fast reactions (high turnover number), ease of use, and continuous reaction capability. It is especially the last property that has been leveraged in a multitude of electrochemical biosensors by using the enzyme to either consume or produce electroactive species over time.

**Figure 9:**
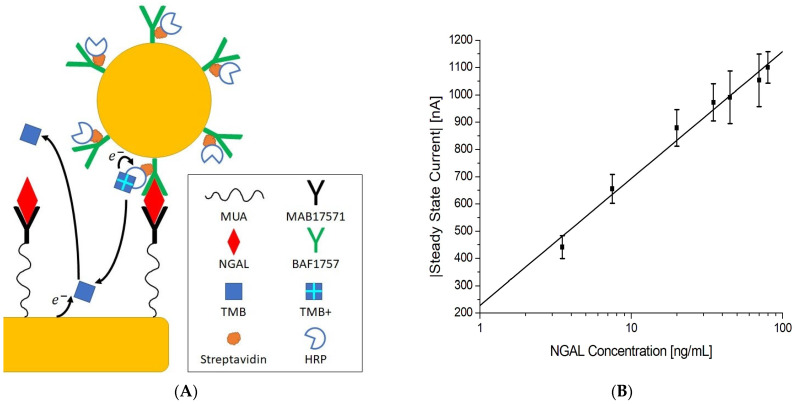
(**A**) Schematic structure of an affinity-based electrochemical sensor. In the presence of analyte (NGAL), AuNP complexes containing secondary antibodies and HRP bind to the electrode surface modified with primary antibodies hence forming a sandwich assay. Electrochemical current generated by reduction of enzymatically produced TMB+ can be used as a signal readout, proportional to the amount of analyte. (**B**) electrochemical response of the sensor to urine samples spiked with different concentration of analyte. [74].

The latter is typically done by having excess substrate so that the enzymatic reaction can be sustained for an extended period, which results in a steady-state response that can be measured by, e.g., chronoamperometry. In addition, it is not uncommon to see biosensor designs make use of enzymes together with nanomaterials, as this typically improves sensitivity and signal response enhancing the overall performance. Pedersen et al. [74] showcased how an HRP enzyme and AuNPs might be used in this fashion. The biosensor consists of a gold SPE that was modified with 11-mercaptoundecanoic acid, and then activated with EDC/NHS chemistry to immobilize the primary antibodies. Afterwards the biosensor was exposed to a urine sample spiked with the target and AuNPs coated with secondary antibody, which HRP was bound to. This formed a sandwich complex between the primary antibody, target, and secondary antibody-coated AuNPs, as illustrated in Figure 9A. Afterwards the sensor was washed and exposed to a solution containing H_2_O_2_ and TMB. This initiates the enzymatic reaction with HRP, which reduces H_2_O_2_ to H_2_O and oxidizes TMB to TMB^+^, where TMB^+^ can be electrochemically reduced back, when in proximity to the electrode surface, generating a current. Therefore, as long as H_2_O_2_ and TMB are in excess, a steady state current can be measured using chronoamperometry, which is linked to the concentration of HRP or more specifically to the target concentration as shown in Figure 9B.

The use of enzymatic labels is not without its shortcomings. One of these is the possible interference from other reagents/chemicals that can inhibit the enzymes’ activity or give rise to a nonspecific signal due to interactions with the electroactive species. Another issue is the enzyme’s limited lifespan, which results from its gradual denaturation over time. This directly impacts the long-term storage capabilities of enzyme-based biosensors. In addition, it is necessary to consider how the enzymatic label is part of the biosensor system. Often this is done by binding the enzyme to a receptor or a nanomaterial, however, this process may adversely affect its activity due to structural changes in the enzyme or by making the active site inaccessible, e.g., the site faces toward the surface of a nanomaterial. As previously mentioned nanozymes could be used as a replacement for enzymes overcoming these challenges, even though some new issues, such as lower specificity, difficulties in mimicking and matching enzymatic activity (lower turnover number), and synthesizing reproduceable batches of nanomaterials (e.g., shape and size) might be introduced [170,186,187]. For a more detailed overview on enzyme-based electrochemical biosensors and how these could be designed see reviews [188,189,190] and for more information on nanozymes we suggest [170,186,187,191].

### 5.4. Redox Probes

**Figure 10:**
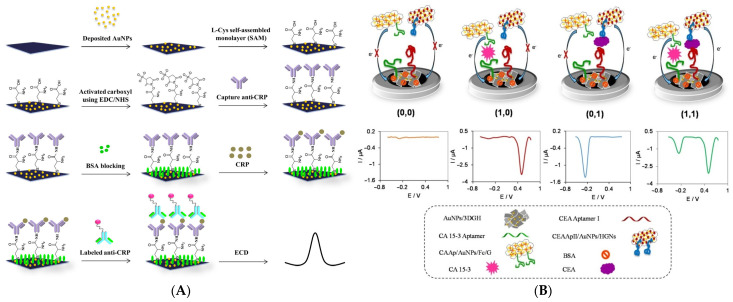
(**A**) Schematic of CRP biosensor build-up and readout from Jampasa et al. [192]. AuNPs are electrodeposited onto a graphene SPE and modified with primary anti-CRP antibody. The detection relies on the amount of anthraquinone-labelled secondary antibody. The redox signal is detected via DPV. (**B**) Schematic representation of electrochemical sensor for multitarget detection by Shekari et al. [193]. A glassy carbon electrode is modified to allow for attachment of two primary aptamers, hence enabling two different sandwich assays, labelled with two distinct redox probes, ferrocene and hemin, on the same electrode. The measurements are performed with DPV which can distinguish the signal from each probe. (reprinted with permission from Elsevier).

As previously mentioned in Section 4 the use of electrochemical techniques together with a redox probe is a combination that has been seen as a staple. Therefore, creating labels that are made of redoxes probes or other electroactive spices is of especial interest. A typical method to introduce the label into the biosensor is by binding the redox probe to a receptor molecule, which can then be part of a sandwich-like configuration. Jampasa et al. [192] made such a design as illustrated in Figure 10A. Initially a graphene SPE was modified using electrodeposition to form AuNPs on the surface, which were exposed to L-cysteine to form a SAM and introduce the carboxyl groups for the immobilization of the first antibody via EDC/NHS chemistry. Afterwards the surface is blocked using bovine serum albumin to decrease nonspecific binding when incubating the target (C-reactive protein or CRP) and anthraquinone-labelled secondary antibody on the biosensor. Based on that design the electrochemical signal, obtained via DPV, will scale depending on the amount of attached redox probe (anthraquinone), which is proportional to the target concentration. One of the unique advantages of using redox labels is that each probe yields a specific signal. Therefore, it is possible to make multiplexing biosensors by labelling different receptors with specific probes, which can then be identified by their distinct electrochemical signal. Shekari et al. [193] demonstrated this principle by using hemin and ferrocene as redox probes that were bound to graphene sheets modified with AuNPs, which the secondary receptor (aptamer) was also bound to. The label complex was used together with a glassy carbon electrode modified with a 3D graphene hydrogel decorated with AuNPs on, which mercaptopropionic acid was bound allowing for the immobilization of the primary receptor (aptamer) via EDC/NHS chemistry. The setup allowed for the individual and simultaneous quantification of two targets based on the amount of each redox probe present in the sandwich-like configuration via DPV as shown in Figure 10B. Although the use of redox probe labels is attractive there are some considerations to keep in mind. One is how the label becomes part of the biosensor: as stated the label is typically bound to a receptor but this process can become quite complex to optimize depending on the method and where the label can bind to the receptor as it may become inactive. Another consideration is the possible presence of interferants that can oxidize/reduce the probe, which would yield a non-specific signal. Lastly it is important to select an appropriate probe. This largely stems from the complex interaction between the probe and the electrode surface as the charge of each, the oxidation/reduction mechanism (inner-sphere/outer-sphere) of the probe, the presence of surface modifications, and the surface sensitivity of the probe all play a role [85,194].

## 6. Discussion/Perspective

The topic of electrochemical biosensors has gathered considerable interest as it has potential applications within numerous fields such as food safety, environmental monitoring, medicine, and biotechnology. As presented in this review there are many aspects to consider when working with electrochemical biosensors that can be used in real-world applications. This involves overcoming challenges regarding stability, reproducibility, sensitivity, sufficient LOD, and complex sample measuring capability.

The first two challenges are often linked to the immobilization method as they are related to the stability of the receptor and electrode surface. The surface and receptors on the sensor need to be stable during measurements and storage, as instability will decrease performance and may yield erroneous outputs. This preferably includes long term stability, typically at least six months, to allow for extended storage of the biosensor. If the sensor becomes nonfunctional within a short time after fabrication it will significantly limit its application potential and useability.

Sensitivity and sufficient LODs are challenges that have been a focal point for researchers for a long time where significant progress has been made to resolve them. The fundamental limitation for this lies within the properties of the receptor. Specifically, it is the affinity/sensitivity of the receptor towards a target and how this is translated into an electrochemical signal that will be limiting the detectable concentration and how easy it is to measure a change in the target concentration. Other ways to improve upon sensitivity and LOD include modifying the electrode surface with nanomaterials improving the electrochemical properties or combing them with a label enhancing the electrochemical readout obtained per binding event.

Lastly it is important for a sensor to function with complex samples, e.g., blood, saliva, urine, sewage, water streams, and beverages. If the sensor cannot measure in a complex sample, it is unsuitable for its intended purpose. Part of the challenge here is that a complex sample will contain many of different constituents (chemicals, proteins, lipids, cells, and microorganisms), which may interfere with the electrochemical performance. To avoid or at least mitigate this matrix effect different approaches have been investigated. The simplest approach is by diluting the sample until the effect becomes negligible, however, this will equivalently dilute the target, which may result in an undetectable concentration. Ideally there should be no need for dilution although this introduces new key parameters to consider as the complex samples will vary in pH, ionic strength, viscosity, and contain possible interferants. Therefore, it is common that samples are partially diluted with a buffer solution to help stabilize the variation of complex samples without overdiluting the target. In addition to this, it is common to block the electrode surface after immobilization before the sensor is exposed to a sample using, e.g., proteins, polymers, or thiols. By blocking these sites beforehand, it will prevent the uncontrolled adsorption of species within the complex sample, which would otherwise impact the reproducibility and sensitivity of the sensor. This is also referred to as the anti-fouling property. Depending on the materials used for blocking it can also change the hydrophobicity/hydrophilicity on the surface, which can further improve the anti-fouling. In addition, the blocking will also help against non-specific binding of the target and label, which will improve the biosensor’s performance.

Another aspect to keep in mind is the electrode geometry. The more commonly used electrodes are either SPEs with a geometry similar to what is presented in Figure 1C or thin-film electrodes with an interdigitated design. For the latter investigations into geometric parameters such as electrode width, gap size, and number of fingers have been conducted, however, the same has not been done for SPEs or at least only to a limited extent. By taking inspiration and knowledge from the thin-film design and applying it to the SPEs it might be possible to improve upon the electrochemical performance of the biosensor without needing sub-micron features.

It is common to include the use of various nanomaterials in biosensors to improve electrochemical properties, sensitivity, selectivity and enable multiplex capabilities. A large number of papers have been published demonstrating the potential feasibility of nanomaterials in electrochemical sensors. However, in-depth investigations of pros and cons of nanomaterials in biosensors are limited and require further research to warrant their use in real-world applications.

When searching for a biosensing system it is essential not only to look for the best LOD, sensitivity, noise level, etc. but to consider the conditions under which this data was obtained. Measurements are often performed only in buffer solution, hence reporting unrealistic results not achievable under real complex sample conditions. On the other hand, the sensors designed to work under such conditions often show worse performance metrics, making them perceived as less attractive. Furthermore, many papers report results based on a limited number of replications or do not provide statistics for the measurements. Repeatability studies where a specific method is tested across multiple laboratories are rare. All this can make comparing different biosensor platforms difficult and time consuming.

Besides the considerations that need to be made for the electrochemical biosensor itself, it is also necessary to consider its application purpose and how it can be used for real-world applications. Here PoC electrochemical biosensors is an application that is highly sought after and many examples in literature can be found that state, they would be suitable for such an application. However, there still has not been observed broad commercialization or widespread usage of such devices. One of the limitations likely lies with the biosensor itself as it still needs to overcome one of more of the challenges presented above, which limits the applicability. Of course, if a sensor is to be commercially available there are some additional requirements that likely must be met such as scalable fabrication methods and low-cost materials. In addition, it is not enough to just have a biosensor, it will have to be integrated into a device that can interact with the sample in a consistent manner and transport it to the sensor surface. This could be achieved using microfluidic systems made using, e.g., polymers or paper to create a simple, robust, and cheap fluidic setup to make low-cost disposable devices. Ideally, such a device should be engineered in a way that allows for one-step measurements that are user-friendly without any complex sample preparation and with minimal involvement of a user. Although there has been success in integrating electrochemical biosensors with fluidic systems it has only shown minimal impact for real-world application.

A fascinating topic that is gaining traction is how machine learning or artificial intelligence can be used together with electrochemical biosensors. Using such tools for data processing and interpretation of raw data could be highly beneficial in removing outliers and finding complex trends that would otherwise go unnoticed, which could improve the biosensor’s sensitivity, accuracy, and precision. Although it is still unclear what benefits there may be, other research areas have already greatly benefited from them, which can be a good indication for the future.

## 7. Conclusions

The field of electrochemical biosensors has continuously gained interest, as is evident by the growing number of publications. The sensors can potentially be used for numerous applications in, e.g., medicine, food safety, water treatment, environmental monitoring, and healthcare offering better stability and sensitivity compared to existing methods.

The most common electrode technology is SPE, where many different electrodes are available on the market, which can be used as a base for PoC electrochemical biosensors. Having so many options allows for a high degree of sensor customization and combined with the option of thin-film electrodes there are even more possibilities. However, cation should be taken when porting particular immobilization protocols from one type of SPE to another even if the electrode material is nominally the same, e.g., gold. The proprietary paste composition together with unspecified thermal treatments leads to different surface properties, which can render a given immobilization strategy ineffective.

The development of blocking methods, surface modifications, interaction of receptors and targets, immobilization of receptors, and optimization of surface properties are crucial areas of research. Extensive optimization in these areas is essential for achieving better performing electrochemical biosensors that are suitable for complex samples.

The introduction of nanomaterials and other labels to electrochemical biosensors offers opportunities for designing new biosensing technologies. This largely stems from the inherent advantages of nanomaterials as they can improve chemical, optical, magnetic, and electrochemical properties of biosensors, opening the path for single-molecule biosensing, multitarget detection, and high throughput arrays. The combination of nanotechnology and PoC electrochemical sensors together with the need for low-cost, mass-produced single-use sensors carries the potential of overturning the current situation where glucose sensors are the only product within electrochemical biosensors to have achieved widespread commercial success.

In conclusion, ongoing research and development in electrochemical biosensors combined with the incorporation of labels and machine learning are pushing towards a new generation of biosensors. That will lead to significant improvements in sensitivity, stability, and real-world application likely to revolutionize a broad range of different fields.

## Data Availability

Data are contained within the article.
